# Clinical, microbiological and genomic characterization of Gram-negative bacteria with dual carbapenemases as identified by rapid molecular testing

**DOI:** 10.1093/jacamr/dlad137

**Published:** 2023-12-30

**Authors:** Ammara Mushtaq, Bremy Alburquerque, Marilyn Chung, Shelcie Fabre, Mitchell J Sullivan, Michael Nowak, Emilia M Sordillo, Jose Polanco, Harm van Bakel, Melissa R Gitman

**Affiliations:** Division of Infectious Diseases, Department of Medicine, Icahn School of Medicine at Mount Sinai, New York, NY 10029, USA; Department of Genetics and Genomic Sciences, Icahn School of Medicine at Mount Sinai, New York, NY 10029, USA; Department of Genetics and Genomic Sciences, Icahn School of Medicine at Mount Sinai, New York, NY 10029, USA; Department of Pathology, Molecular, and Cell Based Medicine, Icahn School of Medicine at Mount Sinai, New York, NY 10029, USA; Department of Genetics and Genomic Sciences, Icahn School of Medicine at Mount Sinai, New York, NY 10029, USA; Department of Pathology, Molecular, and Cell Based Medicine, Icahn School of Medicine at Mount Sinai, New York, NY 10029, USA; Department of Pathology, Molecular, and Cell Based Medicine, Icahn School of Medicine at Mount Sinai, New York, NY 10029, USA; Department of Pathology, Molecular, and Cell Based Medicine, Icahn School of Medicine at Mount Sinai, New York, NY 10029, USA; Department of Microbiology, Icahn School of Medicine at Mount Sinai, New York, NY 10029, USA; Department of Genetics and Genomic Sciences, Icahn School of Medicine at Mount Sinai, New York, NY 10029, USA; Department of Microbiology, Icahn School of Medicine at Mount Sinai, New York, NY 10029, USA; Icahn Genomics Institute, Icahn School of Medicine at Mount Sinai, New York, NY 10029, USA; Department of Pathology, Molecular, and Cell Based Medicine, Icahn School of Medicine at Mount Sinai, New York, NY 10029, USA

## Abstract

**Objective:**

Dual carbapenemase-producing organisms (DCPOs) are an emerging threat that expands the spectrum of antimicrobial resistance. There is limited literature on the clinical and genetic epidemiology of DCPOs.

**Methods:**

DCPO isolates were identified by Xpert^®^ Carba-R PCR testing of routine diagnostic cultures performed from 2018 to 2021 at a New York City health system. WGS was performed by Illumina and/or PacBio. Medical records of patients were reviewed for clinical and epidemiological data.

**Results:**

Twenty-six DCPO isolates were obtained from 13 patients. *Klebsiella pneumoniae* (*n* = 22) was most frequent, followed by *Pseudomonas aeruginosa* (*n* = 2), *Escherichia coli* (*n* = 1) and *Enterobacter cloacae* (*n* = 1). The most common DCPO combination was *bla*_NDM_/*bla*_OXA-48-like_ (*n* = 16). Notably, 1.05% (24/2290) of carbapenem-resistant Enterobacterales isolates were identified as DCPOs. The susceptibility profiles matched the identified resistance genes, except for a *K. pneumoniae* (*bla*_KPC_/*bla*_OXA-48-like_) isolate that was phenotypically susceptible to meropenem. Eleven patients were hospitalized within the year prior to admission, and received antibiotic(s) 1 month prior. Seven patients were originally from outside the USA. Hypertension, kidney disease and diabetes were frequent comorbidities. Death in two cases was attributed to DCPO infection. WGS of eight isolates showed that carbapenemases were located on distinct plasmids, except for one *K. pneumoniae* isolate where NDM and KPC carbapenemases were located on a single IncC-type plasmid backbone.

**Conclusions:**

Here we characterized a series of DCPOs from New York City. Foreign travel, prior hospitalization, antibiotic usage and comorbidities were common among DCPO cases. All carbapenemases were encoded on plasmids, which may facilitate horizontal transfer.

## Introduction

Carbapenem resistance in Gram-negative bacteria occurs due to several mechanisms, which include carbapenemase production, diminished outer membrane permeability, and overexpression of efflux pump, among others.^1^ Production of carbapenemases, however, is the most significant mechanism of resistance since carbapenemases are typically encoded by genes in mobile genetic elements that can be horizontally transferred within species and genera.^2^ Infections with carbapenem-resistant Enterobacterales (CRE) are associated with increased mortality due to limited therapeutic options.^3^ For example, mortality in patients with carbapenemase-producing *Klebsiella pneumoniae* ranges from 18% to 60%.^4^

The threat of carbapenem resistance is exacerbated by the emergence of dual- and multi- carbapenemase-producing organisms (DCPOs and MCPOs, respectively).^2^ Combining multiple carbapenemases can lead to increased resistance to a broader spectrum of β-lactams.^5^ The emergence of most DCPOs/MCPOs is believed to be through stepwise acquisition of additional carbapenemases in an organism that already produces another carbapenemase, rather than transfer of multiple carbapenemases on a single mobile genetic element.^6^

A recent study reported 151 MCPO isolates from the USA from 2010 to 2019, with the incidence of MCPOs reported to be 0.3%, 0.02% and 0.08% for Enterobacterales, *Pseudomonas aeruginosa* and *Acinetobacter baumannii*, respectively.^7^ However, there are limited data available about the clinical risk factors and outcomes of DCPO infection. In this study, we assessed the risk factors and outcomes of patients infected or colonized with DCPOs, as well as the genomic context of carbapenemases within DCPO organisms to better understand acquisition patterns.

## Methods

### Patient selection

A search of the Clinical Microbiology Laboratory at the Mount Sinai Hospital (CML-MSH) laboratory information system identified isolates that tested positive for more than one carbapenemase gene by the Xpert^®^ Carba-R (Cepheid, Sunnyvale, CA, USA). At the time the study was initiated, only one patient was still alive and verbal consent was obtained from the patient’s healthcare proxy. The study protocol was approved by the Mount Sinai School of Medicine Institutional Review Board for the collection and bacterial genome sequencing of discarded clinical specimens (protocol 13-00981 for WGS, and 19-01244 for consenting and chart reviews), as defined by the DHHS regulations.

### Identification of DCPOs

Microbiological cultures were processed using routine procedures at the CML-MSH. Bacteria were identified using MALDI-TOF MS (Bruker Daltonics Inc.). Susceptibility testing was done using VITEK 2^®^ GN 67 (bioMérieux Clinical Diagnostics; 2018–20), MicroScan^®^ NM 53 (Beckman Coulter, Inc; 2020–21) and in some instances, ETEST^®^ (bioMérieux). Susceptibility testing of all isolates was performed for a standard panel of antibiotics. Testing for newer or additional drugs was performed on request by the treating physician if it was clinically indicated, and dependent on availability of the agents in the hospital formulary. If an isolate was phenotypically intermediate or resistant to ertapenem, meropenem or imipenem based on the CLSI breakpoints,^8^ Carba-R or NG-Test ^®^ Carba 5 (NG Biotech, Guipry, France, Hardy Diagnostics) molecular testing was done to identify carbapenemase genes. Carba-R and Carba 5 detect *bla*_KPC_, *bla*_OXA-48-like_, *bla*_NDM_, *bla*_VIM_ and *bla*_IMP_ using PCR testing. All Gram-negatives identified to be positive for ≥2 carbapenemases by Carba-R between 17 July 2018 and 22 March 2021 across the Mount Sinai Health System were included.

The Mount Sinai Pathogen Surveillance Program (MS-PSP) routinely cryopreserves residual CML-MSH isolates from original cultures of clinical and epidemiological significance. Isolates identified to be positive for ≥2 carbapenemase gene were subcultured for WGS. For isolates that had discordance between Carba-R testing of the original CML-MSH subculture and WGS findings of the MS-PSP subculture, we repeated Carba-R testing and performed additional Carba 5 testing on MS-PSP subcultures.

### Chart review

Demographics, vital signs, comorbidities, diagnoses, laboratory data, medications and provider notes of DCPO patients were reviewed. Isolates were recorded as ‘susceptible’ or ‘non-susceptible,’ which included dose-dependent susceptible, intermediate and resistant categories. Data were entered into MS Excel 2017. Charlson comorbidity index (CCI) was calculated for each patient at the time of hospital admission. SOFA score was calculated for patients with non-rectal isolates for the day prior, the day of, and the day after the isolate was collected, and the highest score was charted. Data were analysed by descriptive statistics.

### DNA preparation, WGS and assembly

Single colonies of patient isolates were subcultured to tryptic soy agar plates with 5% sheep blood agar (Thermo Fisher Scientific) at 37°C. After growth overnight, DNA was extracted using the DNeasy Blood & Tissue kit (QIAGEN) following the manufacturer’s guidelines. Illumina libraries were prepared using the DNA Flex Library Kit (Illumina) and sequenced in a paired-end format (2 × 150 nt) on the MiSeq Platform. Draft genomes were assembled using Shovill (https://github.com/tseemann/shovill). Additional sequencing was done on the Pacific Biosciences (PacBio) platform, and curated complete assemblies were generated from HiFi reads using Microbial Assembly (SMRT Link version 10.2) and Flye.^9^ Genomes were annotated using the NCBI Prokaryotic Genome Annotation Pipeline.^10^ Plasmids from these complete assemblies were typed *in silico* using PlasmidFinder^11^ and MOB-suite^12^ and reoriented at the position of replicon sequences using SeqKit.^13^ The small 6.1 kb *bla*_OXA-232_ plasmid from patient #1 was not recovered from the PacBio Flye assembly, but was reconstructed by aligning, trimming and reassembling long reads that mapped to the contig containing *bla*_OXA-232_ in the Illumina assembly from the same isolate. The CARD database was queried with RGI^14^ to evaluate the presence of antibiotic resistance genes (ARGs) in Illumina and/or PacBio genomes. Patient timelines were plotted using R, comparisons between plasmids were made with Easyfig^15^ and circular plasmid maps were generated using SnapGene Viewer (https://www.snapgene.com).

### Comparison with plasmid reference databases

For comparative BLAST searches of plasmids we constructed a reference database from the plasmid genome report (https://ftp.ncbi.nlm.nih.gov/genomes/GENOME_REPORTS/plasmids.txt) and from a query of all bacterial sequence records in the NCBI nucleotide database containing the word ‘plasmid’ in their title as of 27 March 2023. Additional plasmids were obtained from ‘complete’- and ‘chromosome’-level bacterial assemblies from RefSeq or GenBank. Metadata was obtained for all sequences with Entrez Programming Utilities (https://www.ncbi.nlm.nih.gov/books/NBK25501/) and used to remove redundant sequences. Associated metadata was used to retain only accession numbers of complete or circular plasmids.

All genomes and putative plasmid sequences were downloaded using ncbi-genome-download (https://github.com/kblin/ncbi-genome-download/), ncbi-acc-download (https://github.com/kblin/ncbi-acc-download/) or NCBI Datasets (https://www.ncbi.nlm.nih.gov/datasets/) tools. RGI was used to annotate ARGs in the putative plasmids, and the custom list of carbapenemases (ACT-28, AIM, BKC, CTX-M-33, CYM-10, DIM, FLC, FRI, GES, GIM, IMI, IMP, KHM, KPC, LMB, NDM, Nmc, OXA-143, OXA-181, OXA-198, OXA-204, OXA-23, OXA-232, OXA-235, OXA-24, OXA-372, OXA-40, OXA-427, OXA-48, OXA-51, OXA-58, POM, SHV-38, SFC, SFH, SIM, SME, SPM, TMB, VIM) was used to filter for plasmids encoding at least one carbapenemase.

### Data availability

Genome sequences have been deposited in GenBank under BioProject accession number PRJNA470993. Individual accession numbers for sequenced samples are listed in Table S1 (available as Supplementary data at *JAC-AMR* Online).

## Results

### Description of the isolates

Twenty-six DCPO isolates from 13 different patients were identified (Table S1). Twenty-four isolates belonged to the Enterobacterales order, and two were *P. aeruginosa*. During this period, 40 498 Enterobacterales with broad carbapenem susceptibility were reported and 2290 were resistant to at least one carbapenem. A total of 524 of these CRE were positive for a single carbapenemase gene and another 24 were positive for two carbapenemase genes on the Carba-R testing. Thus, the incidence of single CPOs was 22.88% (524/2290) and of DCPOs was 1.05% (24/2290) among CRE isolates. The average number of days from the time of hospital admission to the date of the isolation of a DCPO was 57.5 days (range 0–183 days). Two out of 13 patients (#7 and 10) had a single carbapenemase-positive isolate of the same organism type prior to the identification of DCPO during the same hospital encounter.

Twenty-two DCPO Enterobacterales isolates were *K. pneumoniae*, 14 of which were from rectal swabs (4 with *bla*_NDM_/*bla*_KPC_, 10 with *bla*_NDM_/*bla*_OXA-48-like_), 1 from a penile abscess (*bla*_KPC_/*bla*_OXA-48-like_), 3 from sputum (*bla*_NDM_/*bla*_OXA-48-like_), 1 from trachea (*bla*_NDM_/*bla*_OXA-48-like_), 2 from blood (*bla*_KPC_/*bla*_OXA-48-like_ and *bla*_KPC_/*bla*_NDM_) and 1 from a skin abscess (*bla*_NDM_/*bla*_OXA-48-like_). Twelve *K. pneumoniae* isolates (nine rectal and three sputum) belonged to a single patient (#4) who had received care in Bangladesh. All these isolates were NDM/OXA-48-like co-producers, except for one rectal isolate that was NDM/KPC co-producing. The antibiograms of these isolates were similar, with only varying susceptibility to tigecycline and trimethoprim/sulfamethoxazole. The remaining DCPO Enterobacterales consisted of an *Escherichia coli* isolate from a rectal surveillance culture (*bla*_NDM_/*bla*_OXA-48-like_) and an *Enterobacter cloacae* isolate from a urine culture (*bla*_KPC_/*bla*_OXA-48-like_). The two *P. aeruginosa* isolates were obtained from sputum and rectum of distinct patients and were both positive for *bla*_KPC_/*bla*_OXA-48-like_.

### Antibiogram

DCPO isolates were tested against a panel of antibiotics (Table S1). All 26 DCPO isolates were resistant to penicillins, cephalosporins, ampicillin/sulbactam and piperacillin/tazobactam. All isolates were resistant to the tested carbapenems, except an isolate of *K. pneumoniae* (patient #7), which was resistant to ertapenem and imipenem, but susceptible to meropenem with an MIC of 1 mg/L by VITEK. Seven of the 26 isolates were non-susceptible to all the antibiotics tested, belonging to two different patients (#4 and 9).

Few of the *K. pneumoniae* isolates were susceptible to quinolones, aminoglycosides and trimethoprim/sulfamethoxazole (Table S1). Ceftazidime/avibactam was tested for five isolates, and three were resistant (all with *bla*_NDM_). All four isolates tested for ceftolozane/tazobactam were resistant. Aztreonam was tested for three isolates, and two were resistant (*bla*_KPC_/*bla*_OXA-48-like_ and *bla*_NDM_/*bla*_KPC_).

The *E. coli* and the *E. cloacae* isolates were resistant to ciprofloxacin, levofloxacin and trimethoprim/sulfamethoxazole. The *E. coli* isolate was susceptible to amikacin, but non-susceptible to gentamicin and tobramycin. The *E. cloacae* isolate was susceptible to all three aminoglycosides.

Both *P. aeruginosa* isolates were resistant to ciprofloxacin and levofloxacin, and susceptible to amikacin. One isolate was susceptible to gentamicin. One isolate was tested for tobramycin and ceftazidime/avibactam and was resistant.

### Patient characteristics

Baseline characteristics of the patients are shown in Table 1. Most patients were admitted from home and the mean age was 60.3 years (range 30–94). Eleven patients had previous hospitalization within the last year, and antibiotic exposure within the last month. Seven patients had an ICU stay within 6 months before isolation of the first DCPO. Foreign country of origin was charted for seven patients, who were from Nigeria, Egypt, China, Bangladesh, Guyana, Peru and Yemen. All patients had at least one underlying chronic medical problem. Hypertension, kidney disease and diabetes were the most frequently seen comorbidities. Among patients with rectal isolates (*n* = 7), two had a DCPO isolated from another site following the rectal isolate. Six patients died at the end of their hospitalization, and two were enrolled in palliative care. In two of these cases, death was directly due to the infection from a DCPO (patients #7 and 12).

**Table 1.** Baseline characteristics of patients with Gram-negative DCPOs

Characteristic	Patients (*n*)
Admission location	
Home	9
Nursing home	2
Subacute rehabilitation	2
Age (years)	
≤ 18	0
19–64	6
≥ 65	7
Sex	
Male	9
Female	4
Hospitalized within last year	
Yes	11
No	2
ICU stay during current admission	
Yes^a^	8
No	5
ICU stay within last 6 months of the first DCPO	
Yes	7
No	6
Any antibiotic exposure in last 30 days prior to the current admission	
Yes	11
No	2
Carbapenem exposure in last 30 days^b^	
Yes	4
No	9^b^
Carbapenem-resistant isolate in last 6 months	
Yes	6
No	7
ESBL-positive isolate in last 6 months	
Yes	2
No	11
Foreign travel or origin	
Yes	8
No	5
Comorbidities	
Hypertension	9
Chronic kidney disease or end-stage renal disease	7
Diabetes	6
Hypothyroidism	3
Peripheral vascular disease	3
Coronary artery disease	2
Paraplegia/quadriplegia	2
Atrial fibrillation	2
Chronic suprapubic catheter	2
Heart failure	2
Gastroesophageal reflux disorder	2
Aortic stenosis	2
Hyperlipidaemia	2
Others (COPD, cirrhosis, pulmonary HTN, sickle cell disease, asthma, hepatitis B, diffuse large B-cell lymphoma, non-ischaemic cardiomyopathy, rheumatic fever, traumatic brain injury, HIV, substance use disorder, arthritis, chronic Foley catheter, aortic regurgitation, mitral regurgitation, tricuspid regurgitation, rheumatoid arthritis and obesity)	1 (for each comorbidity)

HTN, hypertension.

^a^Rectal surveillance is routinely performed in ICU patients at our centre, which likely accounts for the higher number of DCPOs seen in ICU.

^b^For patients 5 and 8, chart review revealed that patients were administered antibiotics in the last 30 days, but did not state which antibiotic; they were counted as non-carbapenem.

**Figure 1.**
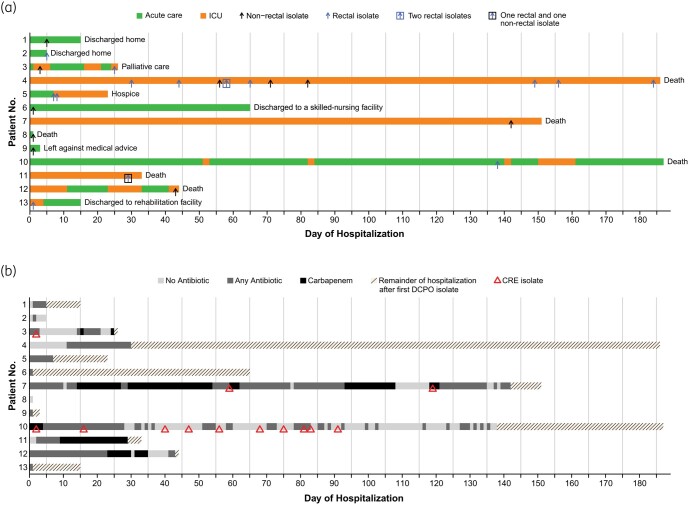
Duration of hospitalization, severity of illness, prior antibiotic exposure and resistant Gram-negative bacteria, and outcomes of patients with dual carbapenemase-positive Gram-negative bacteria. (a) The timeline for each patient from admission up to discharge is shown as horizontal bars with patient location indicated in different colours (legend at top) and outcomes indicated on the right. Dual carbapenemase-producing isolates are indicated by arrows. (b) Timeline of each patient from the time of hospital admission to the first dual carbapenemase-producing isolate, with different colours showing prior antibiotic regimen (legend on top) and triangles showing prior carbapenem-resistant isolates as risk factors.

Characteristics of patients with DCPOs from sites other than rectal surveillance are presented in Table 2. Ten non-rectal isolates were included from eight different patients. The isolates were considered pathogenic in six of those cases by two independent investigators specializing in infectious diseases. The mean CCI score for eight patients at admission was 3, and the mean SOFA score (within 1  day of isolation) of the 10 non-rectal isolates was 7.9. The outcomes are shown in Figure 1(a). Of the eight patients, five died during hospitalization, one was discharged to a skilled-nursing facility, one left against medical advice, and one was discharged home. Figure 1(b) details antibiotic exposure and history of resistant Gram-negative bacteria for each patient from the time of hospital admission to isolation of the first DCPO. Twelve of 13 patients received antibiotic(s) prior to the isolation of the first DCPO during their hospitalization.

**Table 2.** Description of non-rectal DCPOs

Patient	Age/ sex	Pathogen	Source	Carbapenemase gene identified by Carba-R	Admitting diagnoses	CCI at admission	Highest SOFA within 1 day of isolation	Pathogenic	Treated	Antibiotics (MIC, mg/L)	Duration (days)
1	30/F	*K. pneumoniae*	Thigh abscess	*bla*_NDM_, *bla*_OXA-48-like_	Thigh abscess	3	6	Yes	Yes	Tigecycline (2)	14
4	47/M	*K. pneumoniae*	Sputum	*bla*_NDM_, *bla*_OXA-48-like_	Acute exacerbation of heart failure	3	7	No	No	NA	NA
		*K. pneumoniae*	Sputum	*bla*_NDM_, *bla*_OXA-48-like_			8	No	No	NA	NA
		*K. pneumoniae*	Sputum	*bla*_NDM_, *bla*_OXA-48-like_			10	Yes	Yes	Tigecycline (1.5)	14
6	37/M	*P. aeruginosa*	Sputum	*bla*_KPC_, *bla*_OXA-48-like_	Severe sepsis likely due to pneumonia	0	2	Yes	Yes	Ceftazidime/avibactam (2)	7
7	76/M	*K. pneumoniae*	Penile abscess	*bla*_KPC_, *bla*_OXA-48-like_	Urosepsis in setting of colovesical fistula	4	2	Yes	Yes	Meropenem (1)	7
8	94/F	*E. cloacae*	Urine	*bla*_KPC_, *bla*_OXA-48-like_	Sepsis with acute or chronic respiratory failure secondary to pneumonia; congestive heart failure exacerbation	7	12	No	No	NA	NA
9	39/M	*K. pneumoniae*	Blood	*bla*_KPC_, *bla*_OXA-48-like_	Acute osteomyelitis	0	0	Yes	Yes	Minocycline (MIC ≤4; history of anaphylaxis to penicillin and concern of manipulation of IV lines)	2 (patient left against medical advice)
11	55/M	*K. pneumoniae*	Trachea	*bla*_NDM_, *bla*_OXA-48-like_	Severe aortic stenosis	1	18	No	No	NA	NA
12	41/F	*K. pneumoniae*	Blood	*bla*_NDM_, *bla*_KPC_	Necrotizing soft tissue infection	6	14	Yes	Patient died before culture results were available		

M, male; F, female; NA, not applicable.

### Genomic characterization

Thirteen isolates from 11 patients identified by the Carba-R assay in the CML-MSH were available from the MS-PSP biobank for further analysis and were sequenced on the Illumina and/or PacBio platforms. The remaining isolates were not available in the biobank. We confirmed the presence of dual carbapenemases in 9 of the 13 genomes (Table S1). In the remaining four genomes (two *K. pneumoniae*, one *E. cloacae* complex and one *P. aeruginosa*), *bla*_OXA-48-like_ was absent. Repeat Carba-R and Carba 5 testing on the MS-PSP isolates confirmed the WGS results and the discordance with the original CML-MSH test result. To reconstruct the dual carbapenemase resistance loci in their genomic context, we assembled complete genomes for one isolate from each of the eight remaining DCPO patients. All carbapenemases were located on distinct plasmids, except for patient #12, where both carbapenemases were present on a single plasmid (p12.1) (Table 3). Carbapenemase-containing plasmids from the same patient contained distinct replicons (Table 3).

**Table 3.** Carbapenemase-encoding plasmids identified in DCPO isolates

Pt	Organism	MLST type	Carbapenemase gene identified by Carba-R	Plasmid name	Carbapenemase gene identified by PacBio sequencing	Plasmid length (bp)	Plasmid replicon	MOB-suite transferability prediction
1	*K. pneumoniae*	14	*bla*_NDM_, *bla*_OXA-48-like_	p1.1	*bla*_NDM-1_	345 497	IncFIB(pNDM-Mar), IncFIB(pQil), IncFII(K), IncHI1B(pNDM-MAR)	Conjugative
				p1.2	*bla*_OXA-232_	6141	ColKP3	Mobilizable
2	*E. coli*	410	*bla*_NDM_, *bla*_OXA-48-like_	p2.1	*bla*_NDM-5_	95 129	IncFIA, IncFIB(AP001918), IncFII(pAMA1167-NDM-5), IncQ1	Mobilizable
				p2.2	*bla*_OXA-181_	51 473	ColKP3, IncX3	Conjugative
3	*K. pneumoniae*	3515	*bla*_NDM_, *bla*_KPC_	p3.1	*bla*_KPC-3_	57 229	IncN	Conjugative
				p3.2	*bla*_NDM-1_	53 007	IncX3	Conjugative
4	*K. pneumoniae*	147	*bla*_NDM_, *bla*_OXA-48-like_	p4.1	*bla*_NDM-5_	97 919	IncFII	Conjugative
				p4.2	*bla*_OXA-232_	6141	ColKP3	Mobilizable
5	*Klebsiella variicola* (*K. pneumoniae* complex)	971	*bla*_NDM_, *bla*_KPC_	p5.1	*bla*_KPC-5_	315 919	IncHI2, IncHI2A	Conjugative
				p5.2	*bla*_NDM-1_	301 711	IncU^a^	Conjugative
11	*K. pneumoniae*	147	*bla*_NDM_, *bla*_OXA-48-like_	p11.1	*bla*_NDM-5_	155 277	IncFII, IncR	Conjugative
				p11.2	*bla*_OXA-232_	6141	ColKP3	Mobilizable
12	*K. pneumoniae*	409	*bla*_NDM_, *bla*_KPC_	p12.1	*bla*_NDM-1_, *bla*_KPC-3_	205 806	IncC	Conjugative
13	*K. pneumoniae*	395	*bla*_NDM_, *bla*_OXA-48-like_	p13.1	*bla*_NDM-5_	94 578	IncFII	Conjugative
				p13.2	*bla*_OXA-232_	6141	ColKP3	Mobilizable

^a^Replicon identified with MOB-suite instead of PlasmidFinder using default parameters.

**Figure 2.**
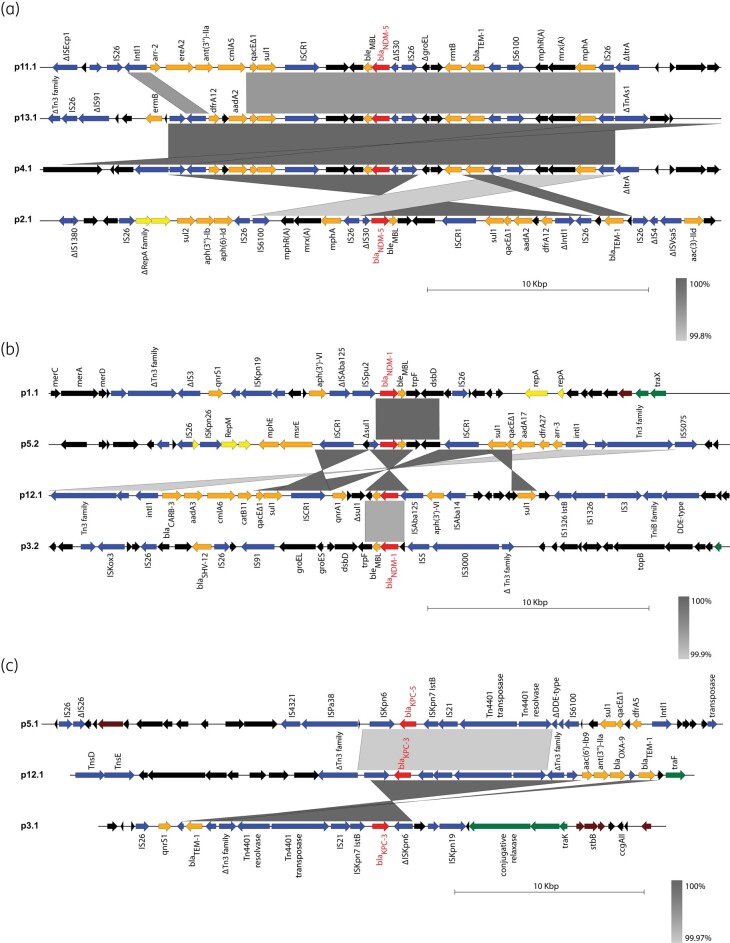
Comparison of loci containing *bla*_NDM-5_, *bla*_NDM-1_ and *bla*_KPC_ shows conserved regions surrounded by variable mobile elements and ARGs. The loci containing (a) *bla*_NDM-5_, (b) *bla*_NDM-1_ or (c) *bla*_KPC_ in each plasmid were compared with one another using Easyfig. Sequences ∼15 kb upstream and downstream from each carbapenemase were included for comparison. Regions of similarity at least 1000 bp in length are highlighted in different shades of grey, based on the percent identities indicated in the legend for each panel. Arrows represent annotated coding sequences, and annotations for some of these sequences are included in the maps. Genes encoding carbapenemases are shown in red, other antibiotic resistance in orange, mobile elements/recombination in blue, plasmid transfer in green, plasmid replication in yellow, plasmid maintenance/antirestriction in brown, and all other coding sequences in black.

Most DCPO genomes contained a combination of *bla*_NDM_ and *bla*_OXA-48-like_ carbapenemases (*bla*_OXA-181_ or *bla*_OXA-232_). Three of the four *bla*_NDM-5_ plasmids (p4.1, p11.1, p13.1) contained IncFII or IncFII/IncR replicons and shared large segments, suggesting common ancestry (Figure S1a). The *bla*_NDM-5_ loci in all four plasmids were associated with class 1 integrons, multiple IS*26* and IS*CR1* insertion sequences (Figure 2a) and a variety of ARGs such as *aadA2*, *ant(3′)-IIa*, *arr-2*, *bla*_TEM-1_, *ble*_MBL_, *cmlA*, *dfrA12*, *ereA2*, *erm*(B), *mph*(A), *qacEΔ1*, *rmtB* and *sul1*. The four *bla*_OXA-232_ plasmids, containing ColKP3 replicons, were nearly identical to each other (Figure S2a). The single *bla*_OXA-181_ carbapenemase was found on a 51 473 bp ColKP3/IncX3 plasmid in the *E. coli* isolate from patient #2, which also encoded the QnrS1 fluoroquinolone resistance protein (Figure S2b).

The three remaining DCPO isolates contained a combination of *bla*_NDM-1_ and *bla*_KPC_ (*bla*_KPC-3_ or *bla*_KPC-5_). The *bla*_NDM-1_ plasmids contained the IncX3, IncU or IncC replicons (Figure S1b, Table 3) but shared a small, conserved region with full length or truncated versions of *bla*_NDM-1_, *ble*_MBL_, *trpf* and *dsbD* (Figure 2b) surrounded by different sets of ARGs in each plasmid (Figure 2b). The *bla*_KPC_ plasmids were also distinct (Figure S1c) but shared a Tn*4401* transposon containing IS*Kpn7-bla*_KPC_*-*IS*Kpn6* (Figure 2c). Nearby ARGs included *qnrS1* and *bla*_TEM-1_ in p3.1; *sul1*, *qacEΔ1* and *dfrA5* in p5.1; and *aac(6′)-Ib9*, *ant(3′′)-IIa*, *bla*_OXA-9_ and *bla*_TEM-1_ in p12.1.

**Figure 3.**
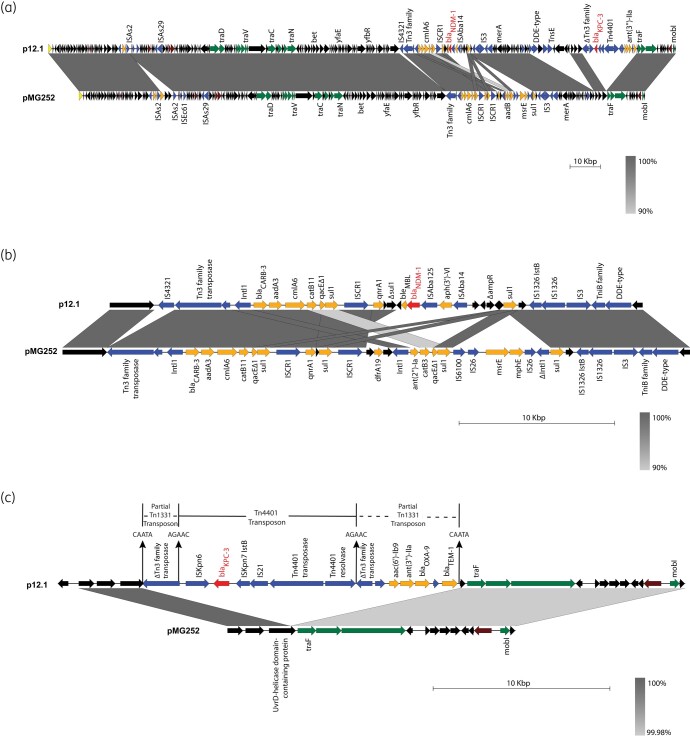
Plasmid carrying both *bla*_NDM-1_ and *bla*_KPC-3_ shows significant similarity to pMG252. (a) The plasmid carrying both *bla*_NDM-1_ and *bla*_KPC-3_ was compared with the pMG252 plasmid in *E. coli* J53 (GenBank accession MK638972.1) using Easyfig. Regions of similarity at least 1000 bp in length are highlighted in different shades of grey, based on the percent identities indicated in the legend for each panel. Arrows represent annotated coding sequences, and annotations for some of these sequences are included in the maps. Genes encoding carbapenemases are shown in red, other antibiotic resistance in orange, mobile elements/recombination in blue, plasmid transfer in green, plasmid replication in yellow, plasmid maintenance/antirestriction in brown, and all other coding sequences in black. Zoomed-in versions of the loci surrounding (b) *bla*_NDM-1_ and (c) *bla*_KPC-3_ in each of the plasmids are also included. Direct repeats (CAATA or AGAAC) flanking the inverted repeats of the interrupted Tn*1331* or Tn*4401* transposon are depicted with arrows above the plasmid map in (c).

Lastly, we analysed the 205 806 bp IncC-type plasmid p12.1, harbouring both *bla*_NDM-1_ and *bla*_KPC-3_. Although a BLAST search of the NCBI nucleotide database revealed no identical matches, p12.1 shared large segments with pMG252, the first plasmid documented to confer quinolone resistance.^16,17^ (Figure 3a). Compared with pMG252, a 15 kb region in an antibiotic resistance island was replaced by an ∼7 kb region in plasmid p12.1 that contained *bla*_NDM-1_ (Figure 3b). A comparative analysis of the *bla*_KPC-3_ locus suggests that p12.1 acquired *bla*_KPC-3_ via a Tn*4401* transposition event (Figure 3c). This transposon inserted into a preexisting Tn*1331* transposon that had already interrupted a UvrD helicase upstream of *traF* (Figure 3c). A BLAST search revealed multiple plasmid hits with at least 75% coverage of p12.1 that encoded individual *bla*_NDM_, *bla*_KPC_ or *bla*_OXA-427_ carbapenemases, suggesting p12.1 may have evolved through sequential carbapenemase acquisition. We created a database of all complete or circular bacterial plasmids in NCBI encoding carbapenemases as of 27 March 2023 and found that out of 5081 carbapenemase-encoding plasmids, only 56 (1.1%) encoded two different types of carbapenemases. Four of these plasmids contained a combination of *bla*_NDM_ and *bla*_KPC_, but did not share the same backbone as p12.1. Thus, the presence of dual carbapenemases on a single plasmid backbone remains a rare occurrence.

## Discussion

The presence of multiple carbapenemases in Gram-negative bacteria in hospitalized patients, as seen in this study, is concerning for three reasons. First, the combined spectrum of β-lactam hydrolysis in DCPOs is wider than in isolates producing a single carbapenemase, thereby further compromising already limited therapeutic options. NDM and OXA-48, for example, do not hydrolyse aztreonam, but when present together with KPC, the hydrolytic spectrum widens to include aztreonam.^2^ Second, most carbapenemase genes in DCPOs are located within mobile genetic elements, which may facilitate horizontal gene transfer, and lead to multispecies dissemination of DCPOs and enrichment of the resistance gene pool. Lastly, the presence of multiple carbapenemases threatens the utility of even newer antibiotics. Therefore, the antibiotic development pipeline should continue to expand to cope with new resistance mechanism combinations.

Interestingly, we found one patient with a meropenem-susceptible, but ertapenem- and imipenem-resistant, *bla*_KPC_/*bla*_OXA-48-like_-positive *K. pneumoniae* isolate. Carbapenems for treatment of CPOs have previously been discussed.^18^ OXA-48 is considered a ‘weak’ carbapenemase, and in the absence of other enzymes it does not hydrolyse ceftazidime or aztreonam.^18^ Livermore *et al*.^18^ have previously shown that the carbapenem MICs conferred were in the lowest range for OXA-48, and highest for NDM. In fact, over half of OXA-48-producing isolates were considered susceptible to meropenem at the CLSI breakpoint of 1 mg/L. Clinical use of carbapenems for OXA-48-producing pathogens, however, has shown poor outcomes, even at low MICs.^17^ The bacteraemia patient with *bla*_KPC_/*bla*_OXA-48-like_-positive and meropenem-susceptible *K. pneumoniae* was treated with meropenem but died of infection from the dual carbapenemase-producing *K. pneumoniae*. The presence of an inoperable colovesical fistula suggested that a lack of source control contributed to the patient’s demise.

Of the 13 isolates sequenced, 9 were found to contain dual carbapenemase genes that matched the clinical test profile. Interestingly, the discordance only arose in cases of OXA-48-like carbapenemases, suggesting potential instability of a *bla*_OXA-48-like_ plasmid. Thus, one potential explanation for the discrepancy between the testing and genomics data is the presence of a heterogeneous population of OXA-48-like-producing and non-producing bacteria in the clinical specimen^19–22^ that resulted in the selection of subclones with different susceptibility profiles for Carba-R testing and sequencing. Alternatively, it is possible that the isolate lost one of the carbapenemases during a subcloning step. Lastly, false-positive Carba-R results are rare but have been reported in a small percentage of cases.^23^

Most DCPO isolates encoded carbapenemases on distinct plasmids, suggesting that sequential acquisition of plasmids is the most likely mechanism for acquiring multiple carbapenemases. However, in one of the patients whose death was directly attributed to the DCPO infection, we observed both *bla*_NDM_ and *bla*_KPC_ on a single IncC-type plasmid (p12.1) that shares significant similarity to pMG252. Compared with pMG252, ARG changes in p12.1 were observed in an antibiotic resistance island that acquired *bla*_NDM-1_. The island in pMG252 contained multiple copies of *sul1* that may have mediated the ARG recombination events, as proposed in other reports involving *sul1* loci.^24,25^ In addition, acquisition of *bla*_KPC-3_ occurred further downstream in p12.1 via a Tn*4401* transposition event. IncC conjugative plasmids are already prevalent among Gram-negatives, and their broad host range has facilitated the spread of ARGs such as carbapenemases across multiple species.^26–30^ Thus, the insertion of multiple carbapenemases in IncC-type plasmids increases the risk for further dissemination of DCPOs with high-level carbapenemase resistance. Further work is needed to confirm whether p12.1 can be readily transferred to other organisms.

Our work had limitations inherent to a retrospective study. Only some of the clinical isolates were available for sequencing. Our study also did not capture clinical information (e.g. antibiotics, hospitalization) outside our health system where patients may have received treatment. Although we did not detect obvious loss-of-function mutations (e.g. stop-gain, frameshift) in the DCPO genes, we did not perform phenotypic testing of the DCPO isolates to confirm that the DCPO genes were functional. With the advent of rapid molecular diagnostics, appropriate management of patients that test positive for genotypic resistance targets but are phenotypically susceptible needs to be considered.^31^ This is a single-centre study reporting a limited collection of DCPOs. Larger studies are needed to adequately assess the risk factors and clinical outcomes of such infections.

In conclusion, we report a collection of DCPOs from New York City. Infectious diseases physicians should be aware of this threat, as our study shows high mortality in patients infected or colonized with DCPOs. Further research into appropriate management of infections caused by DCPOs is needed. In a critically ill patient with a bacterial syndrome and in the presence of appropriate epidemiological links, combination antibiotic agents should be considered empirically to cover for the possibility of DCPOs. There should be ongoing surveillance of DCPOs, and of plasmids encoding more than one class of carbapenemases. Cases where *bla*_OXA-48-like_ was initially detected by Carba-R, but not on WGS merit further investigation for the possible hypotheses discussed above.

## Supplementary Material

dlad137_Supplementary_Data

## Supplementary data

Figures S1 and S2 and Table S1 are available as Supplementary data at *JAC-AMR* Online.
