# Supplementary material for: Clinical, microbiological and genomic characterization of Gram-negative bacteria with dual carbapenemases as identified by rapid molecular testing
Source: JAC Antimicrob Resist. 2023 Dec 30;6(1):dlad137. doi: 10.1093/jacamr/dlad137 (PMC10757448; doi:10.1093/jacamr/dlad137)
Supplement: dlad137_Supplementary_Data [file dlad137_supplementary_data.zip › Supplementary_Figures.pdf]

Figure S1

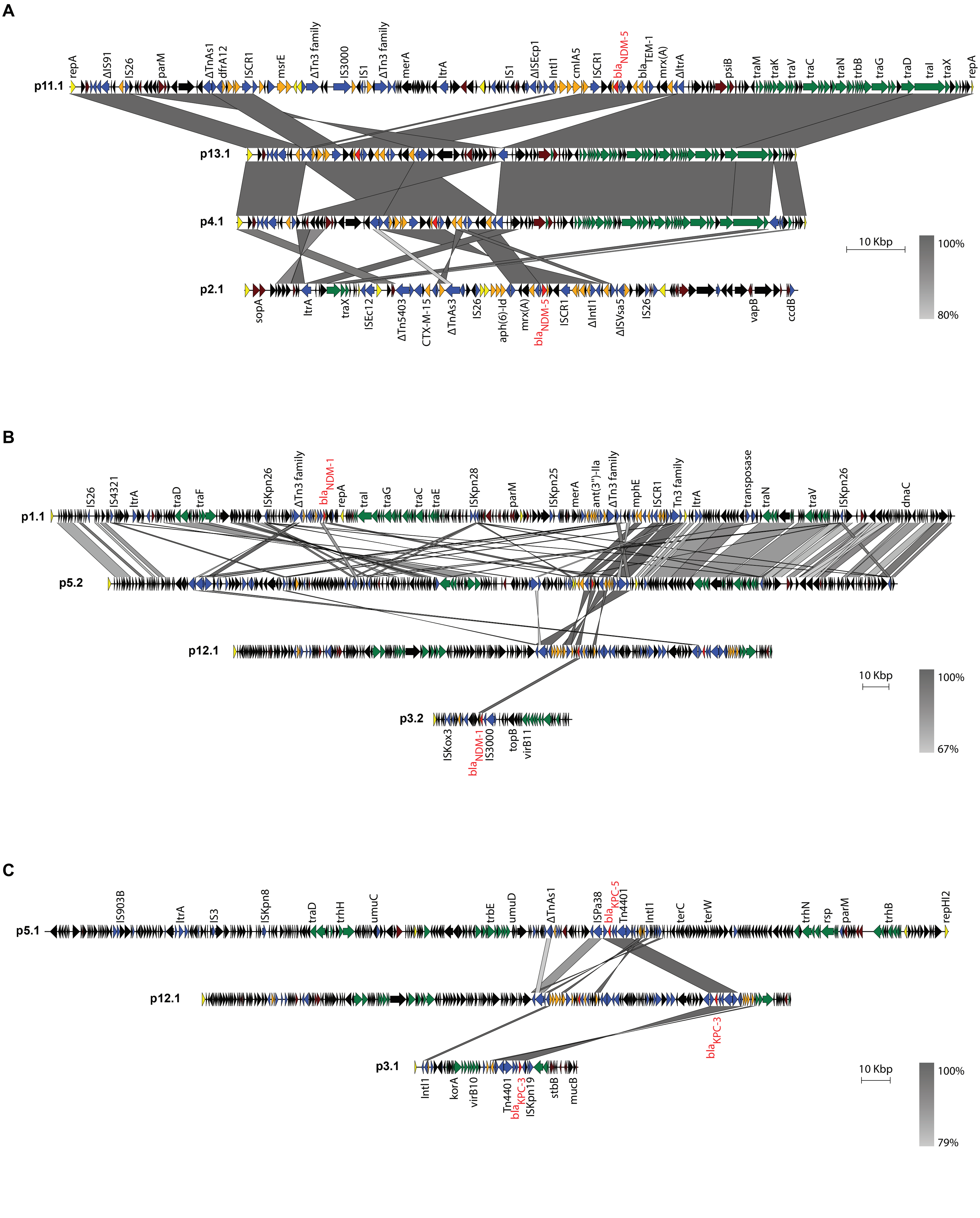
A
repA
ΔIS91
IS26
parM
ΔTnAs1
dfrA12
ISCR1
msrE
ΔTn3 family
IS3000
IS1
ΔTn3 family
merA
ltrA
IS1
ΔISEcp1
IntI1
cmlA5
ISCR1
blaNDM-5
blaTEM-1
mrx(A)
ΔltrA
psiB
traM
traK
traV
traC
traN
trbB
traG
traD
traI
traX
repA
p11.1
p13.1
p4.1
p2.1
sopA
ltrA
traX
ISEc12
ΔTn5403
CTX-M-15
ΔTnAs3
IS26
aph(6)-Id
mrx(A)
blaNDM-5
ISCR1
ΔIntI1
ΔISVsa5
IS26
vapB
ccdB
10 Kbp
100%
80%
B
IS26
IS4321
ltrA
traD
traF
ISKpn26
ΔTn3 family
blaNDM-1
repA
traI
traG
traC
traE
ISKpn28
parM
ISKpn25
merA
ant(3'')-IIa
ΔTn3 family
mphE
ISCR1
Tn3 family
ltrA
transposase
traN
traV
ISKpn26
dnaC
p1.1
p5.2
p12.1
p3.2
ISKox3
blaNDM-1
IS3000
topB
virB11
10 Kbp
100%
67%
C
IS903B
ltrA
IS3
ISKpn8
traD
trhH
umuC
trbE
umuD
ΔTnAs1
ISPa38
blaKPC-5
Tn4401
IntI1
terC
terW
trhN
rsp
parM
trhB
repHI2
p5.1
p12.1
blaKPC-3
p3.1
IntI1
korA
virB10
Tn4401
blaKPC-3
ISKpn19
stbB
mucB
10 Kbp
100%
79%

Figure S2

A

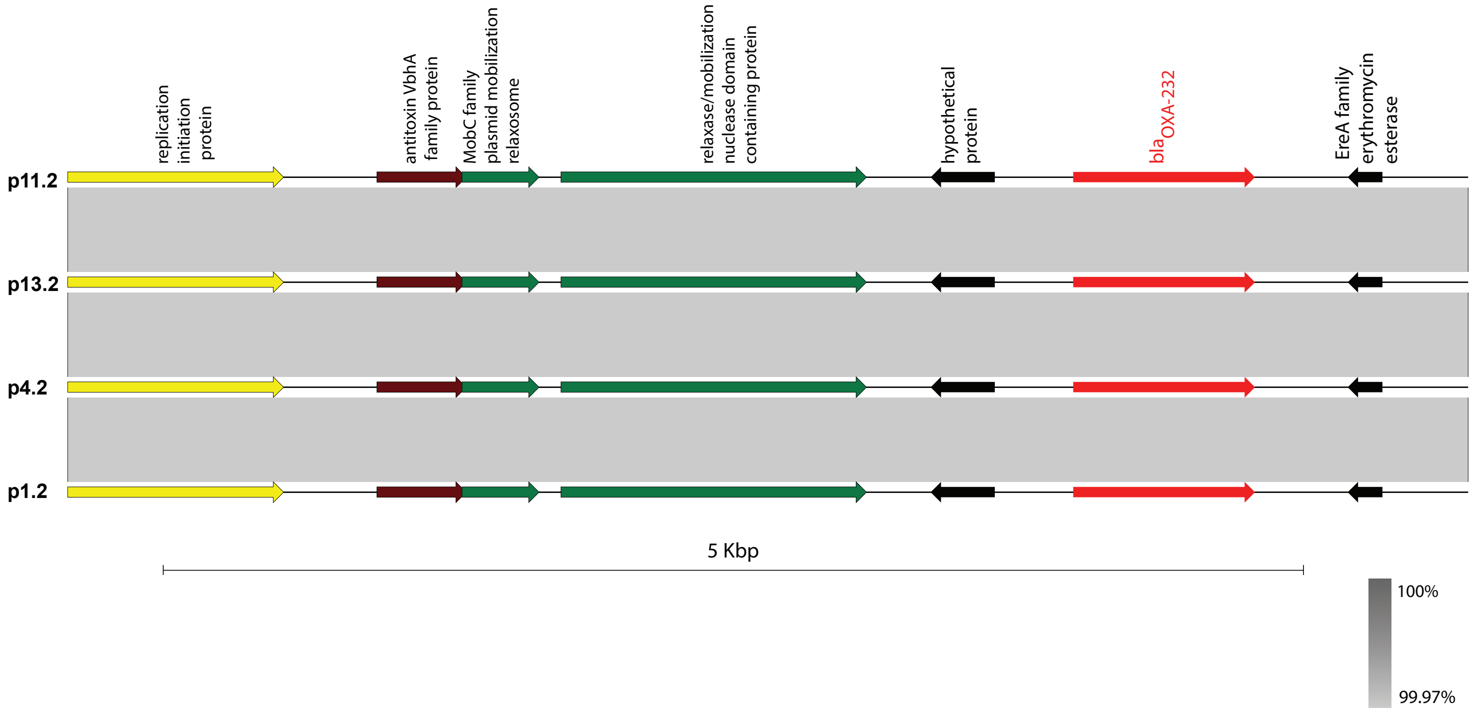

replication initiation protein
antitoxin VbhA family protein
MobC family plasmid mobilization relaxosome
relaxase/mobilization nuclease domain containing protein
hypothetical protein
blaOXA-232
EreA family erythromycin esterase
p11.2
p13.2
p4.2
p1.2
5 Kbp
100%
99.97%

B

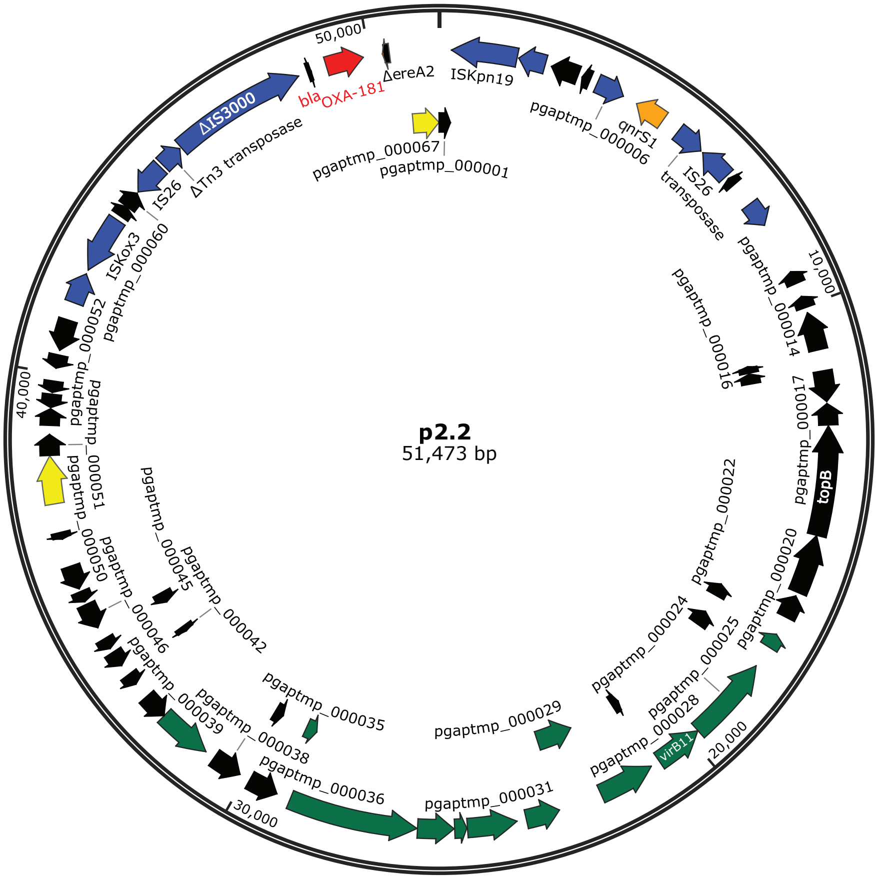

p2.2
51,473 bp
blaOXA-181
ΔereA2
ISKpn19
pgaptmp_000006
qnrS1
IS26 transposase
pgaptmp_000014
pgaptmp_000016
pgaptmp_000017
topB
pgaptmp_000020
pgaptmp_000022
pgaptmp_000024
pgaptmp_000025
pgaptmp_000028
virB11
pgaptmp_000029
pgaptmp_000031
pgaptmp_000035
pgaptmp_000036
pgaptmp_000038
pgaptmp_000039
pgaptmp_000042
pgaptmp_000045
pgaptmp_000046
pgaptmp_000050
pgaptmp_000051
pgaptmp_000052
pgaptmp_000060
ISKox3
IS26
ΔTn3 transposase
ΔIS3000
pgaptmp_000067
pgaptmp_000001
10,000
20,000
30,000
40,000
50,000

SUPPLEMENTARY FIGURE LEGENDS

Supplementary Figure 1. Comparison of *bla*$_{NDM-5}$, *bla*$_{NDM-1}$, and *bla*$_{KPC}$ plasmids from dual carbapenemase producing organisms. Plasmids encoding for either (A) *bla*$_{NDM-5}$, (B) *bla*$_{NDM-1}$, or (C) *bla*$_{KPC}$ were compared to one another using EasyFig. Regions of similarity at least 1000 bp in length between plasmids are highlighted in different shades of gray, based on the percent identities indicated in the legend for each panel. Arrows represent annotated coding sequences, and annotations for some of these sequences are included in the maps. Genes encoding carbapenemases are shown in red, other antibiotic resistance in orange, mobile elements / recombination in blue, plasmid transfer in green, plasmid replication in yellow, plasmid maintenance / antirestriction in brown, and all other coding sequences in black.

Supplementary Figure 2. Maps of plasmids encoding for *bla*$_{OXA-232}$ or *bla*$_{OXA-181}$. (A) Comparison of the four *bla*$_{OXA-232}$ plasmids generated with EasyFig. Regions of similarity at least 1000 bp in length are highlighted in gray, showing that these plasmids are almost identical. Arrows represent annotated coding sequences, with genes encoding plasmid replication in yellow, plasmid maintenance in brown, plasmid transfer in green, carbapenemase in red, and all other coding sequences in black. (B) Genetic map of the only *bla*$_{OXA-181}$ plasmid analyzed in this study, generated using SnapGene. Color-coding of coding regions is the same as in (A), along with mobile element / recombination genes in blue, and the antibiotic resistance gene *qnrS1* highlighted in orange.
